# Extrasynaptic exocytosis and its mechanisms: a source of molecules mediating volume transmission in the nervous system

**DOI:** 10.3389/fphys.2012.00319

**Published:** 2012-09-04

**Authors:** Citlali Trueta, Francisco F. De-Miguel

**Affiliations:** ^1^Departamento de Neurofisiología, Instituto Nacional de Psiquiatría Ramón de la Fuente MuñizMéxico, D.F., México; ^2^Instituto de Fisiología Celular, Universidad Nacional Autónoma de MéxicoMéxico, D.F., México

**Keywords:** exocytosis, extrasynaptic, serotonin, somatic exocytosis, volume transmission, mechanisms of extrasynaptic exocytosis

## Abstract

We review the evidence of exocytosis from extrasynaptic sites in the soma, dendrites, and axonal varicosities of central and peripheral neurons of vertebrates and invertebrates, with emphasis on somatic exocytosis, and how it contributes to signaling in the nervous system. The finding of secretory vesicles in extrasynaptic sites of neurons, the presence of signaling molecules (namely transmitters or peptides) in the extracellular space outside synaptic clefts, and the mismatch between exocytosis sites and the location of receptors for these molecules in neurons and glial cells, have long suggested that in addition to synaptic communication, transmitters are released, and act extrasynaptically. The catalog of these molecules includes low molecular weight transmitters such as monoamines, acetylcholine, glutamate, gama-aminobutiric acid (GABA), adenosine-5-triphosphate (ATP), and a list of peptides including substance P, brain-derived neurotrophic factor (BDNF), and oxytocin. By comparing the mechanisms of extrasynaptic exocytosis of different signaling molecules by various neuron types we show that it is a widespread mechanism for communication in the nervous system that uses certain common mechanisms, which are different from those of synaptic exocytosis but similar to those of exocytosis from excitable endocrine cells. Somatic exocytosis has been measured directly in different neuron types. It starts after high-frequency electrical activity or long experimental depolarizations and may continue for several minutes after the end of stimulation. Activation of L-type calcium channels, calcium release from intracellular stores and vesicle transport towards the plasma membrane couple excitation and exocytosis from small clear or large dense core vesicles in release sites lacking postsynaptic counterparts. The presence of synaptic and extrasynaptic exocytosis endows individual neurons with a wide variety of time- and space-dependent communication possibilities. Extrasynaptic exocytosis may be the major source of signaling molecules producing volume transmission and by doing so may be part of a long duration signaling mode in the nervous system.

## Introduction

Communication in the nervous system is classically known to occur at synapses, where a neurotransmitter released from the presynaptic active zone reaches the postsynaptic membrane and produces a local synaptic potential. The millisecond time scale of these responses allows neuronal circuits to produce rapid behavioral responses, from relatively simple ones, like an avoidance reaction to a warm surface, to others requiring incredible amounts of computation in extremely short periods of time, such as playing tennis at high speed, in which the location of the ball, its speed, trajectory, force, and direction must be calculated in less than a second to hit the ball back and send it to the right place. However, the nervous system also possesses the parallel capacity of changing its responses for very long periods of time, lasting from seconds to days. For example, mood states may change the quality of the tennis player performance by modulating the input–output relationships of the brain. Events lasting for seconds or less may change the responses of our brain circuits for hours or longer. How does this happen?

The discovery of extrasynaptic receptors by Miledi (1960), was followed by observations by Dun and Minota (1982) of peripheral neuronal responses that could be attributed to somatic exocytosis of signaling molecules upon electrical stimulation. In addition, the discovery of extracellular concentrations of monoamines at a distance from synaptic endings, and the mismatch between peptide exocytosis sites and receptors, led Fuxe and colleagues to propose volume transmission as a way of communication in the nervous system, parallel to that of hard-wired circuits (Agnati et al., 1986a,b; Fuxe et al., 2012; see also Fuxe et al., in this issue). The presence of extrasynaptic receptors for acetylcholine, glutamate, and gama-aminobutiric acid (GABA) in vertebrate (for review see Vizi et al., 2010) and invertebrate (see for example, Sargent et al., 1977) neurons and the detection of these transmitters in the extracellular fluid in concentrations capable of activating their receptors suggest that the “classical” low molecular weight neurotransmitters previously thought to act exclusively on synapses, also participate in extrasynaptic communication. In fact, several functions for extrasynaptic communication through these transmitters have been demonstrated in different areas of the central nervous system.

However the source of these molecules in the extracellular space was not clear, and while it was initially thought that it was caused by transmitter spillover from synaptic release sites, the amount of transmitter released from presynaptic endings and their local degradation or uptake makes it difficult to expect that these molecules have a synaptic origin.

Morphological evidence as to the presence of small clear and large dense core vesicles in the soma, axonal varicosities, and dendrites and of dense core vesicles in the periphery of synapses in the absence of postsynaptic counterparts, suggested that transmitters and peptides could also be released extrasynaptically. In the past fifteen years a variety of studies applying diverse techniques have provided direct evidence that serotonin, dopamine, noradrenaline, adenosine-5-triphosphate (ATP), and peptides such as substance P, brain-derived neurotrophic factor (BDNF), or oxytocin, are released by exocytosis from extrasynaptic sites by central and peripheral neurons of invertebrates and vertebrates. These molecules activate mainly metabotropic receptors that exert indirect and therefore slower effects than those of synaptic transmitters acting on ionotropic receptors. This already is sufficient to expand the timing of neuronal communication, and in addition to the diffusion and permanence of transmitter molecules in the extracellular space, explains the long time course of modulation mediated by volume transmission. Acetylcholine, glutamate and GABA have now been added to the list of transmitters acting extrasynaptically.

Extrasynaptic release of low molecular weight transmitters occurs through different mechanisms, including exocytosis, the reversal of transporter proteins, release through pores or diffusion through the plasma membrane. In this review we will focus on extrasynaptic exocytosis of different signaling molecules and its mechanisms. We will start this review with the evidence for extrasynaptic exocytosis of serotonin for two reasons. The first is that many of the mechanisms underlying its exocytosis from the soma have now been studied with detail and thus, it serves as a reference point for the search for general principles. The second is that many of its extrasynaptic effects have been demonstrated experimentally.

## Serotonin

5-hydroxytriptamine (5-HT), also known as serotonin because of the first function described for it, is a monoamine involved in the regulation of multiple functions including sleep, circadian rhythms, digestion, hormone secretion, learning, and the generation of central rhythmic locomotory, respiratory, masticatory and pyloric patterns (McCall and Aghajanian, 1979; Prosser et al., 1990; Raleigh et al., 1991; Jacobs and Azmitia, 1992; Jacobs and Fornal, 1993; White et al., 1996; Weiger, 1997; Hull et al., 1999; Kravitz, 2000; Richards et al., 2003; Cruz-Bermúdez and Marder, 2007). Alterations in the serotonergic system in humans lead to psychiatric disorders such as depression, anxiety, feeding, or sleep disorders or schizophrenia (Charney, 1998; Arango et al., 2002; Brieden et al., 2002; Durant et al., 2010).

### Morphology of serotonergic systems in relation to extrasynaptic exocytosis

A striking but conserved characteristic of serotonergic systems is that relatively small numbers of neurons accomplish multiple functions. While the serotonergic system in a leech intermediate ganglion is composed by a network of three pairs plus one single neuron (out of 400 total neurons), in mammals there are only between 9000 (in rodents) and 90,000 (in humans; Underwood et al., 1999) serotonergic neurons (accounting to a proportion of 1:1,000,000 of the total neurons in the human brain), whose somata are located in the raphe brainstem nuclei, (Dahlström and Fuxe, 1964; Jacobs and Azmitia, 1992).

Serotonergic projections in vertebrates and invertebrates branch profusely and have complex innervations to virtually all areas of the central nervous system, sometimes forming synapses, while others forming varicosities that contain clear and/or dense core vesicles but lacking postsynaptic counterparts (Descarries and Mechawar, 2000; reviewed by Bunin and Wightman, 1999). Serotonergic axons innervating the ventral horns of the spinal cord (Kiehn et al., 1992; Alvarez et al., 1998) or the substantia nigra reticulata (Moukhles et al., 1997) establish mostly synaptic contacts with well-defined target neurons; however in dorsal horns of the spinal cord (Ridet et al., 1993) and in the nucleus accumbens (Van Bockstaele and Pickel, 1993), nearly 60% of the 5-HT terminals do not form synapses. Axons originating in different mammalian nuclei and projecting to the same brain area may have different connection patterns. For example, in the hippocampus, varicosities of fibers arriving from the median raphe form direct synapses with interneurons (Freund and Buzsáki, 1996; Varga et al., 2009), whereas fibers originating in the dorsal raphe form varicosities but rarely synapses (Kosofsky and Molliver, 1987). Recurrent axon collaterals ending in the dorsal raphe contain both synaptic and non-synaptic endings (Chazal and Ralston, 1987).

The dendrites of serotonergic neurons in the dorsal raphe nucleus also contain serotonin in small clear and large dense-core vesicles (Kapadia et al., 1985; Liposits et al., 1985; Chazal and Ralston, 1987). These vesicles can be densely packed in clusters, some of which are located at dendro-dendritic synapses with serotonergic or other types of neurons. Other vesicles are not associated with the characteristic presynaptic active zone specializations, and thus seem to be suitable for extrasynaptic exocytosis. In the somata of serotonergic neurons of leech and rat, visualized by electron microscopy (Coggeshall, 1972; Trueta et al., 2004, 2012) or 3-photon microscopy (Kaushalya et al., 2008a) serotonin accumulates in multivesicular structures, which in leech Retzius neurons have been clearly identified as clusters of dense core vesicles (Trueta et al., 2012). In fact, the amount of serotonin in the somatic area of raphe neurons is quantitatively comparable to that in brain areas with dense serotonergic innervation (Kaushalya et al., 2008b).

All this morphological evidence suggests that serotonin is not only released from presynaptic terminals, but also from extrasynaptic sites in axons, dendrites, and somata.

### Indirect evidence of extrasynaptic serotonin exocytosis

Morphological evidences of extrasynaptic exocytosis sites agree with serotonin measurements in the extracellular fluid. The serotonin concentration and, more importantly, its dynamic changes in response to stimulation, have been monitored by fast-scan cyclic voltammetry in several areas of the central nervous system of vertebrates and invertebrates. Since the size of carbon fiber electrodes used for voltammetry is much bigger than the synaptic clefts, it is generally accepted that these measurements reflect the serotonin concentration in extrasynaptic sites. Extracellular serotonin has been measured by amperometry in the neuropile of the *Aplysia* central nervous system (Marinesco and Carew, 2002), where it modulates synaptic plasticity and simple learning (Marinesco et al., 2006). In mammalian central nervous systems, extrasynaptic serotonin has been detected both in regions with primarily synaptic connections, and in regions where exocytosis seems to be extrasynaptic. In the substantia nigra reticulata (Bunin and Wightman, 1998) or the spinal cord (Hentall et al., 2006), which have serotonergic synaptic terminals, 5-HT has been readily detected in response to single stimulation pulses. In these regions the amount of serotonin molecules released following single impulses is smaller than the amount of receptors and transporters, but its detection is not affected by reuptake inhibitors or receptor antagonists, suggesting that although exocytosis occurs mainly from synaptic terminals, transporters are localized extrasynaptically, thus allowing serotonin spillover (Bunin and Wightman, 1998). On the other hand, extracellular serotonin has also been measured in brain regions such as the dorsal raphe or the hippocampus, which lack synaptic contacts, thus supporting that release occurs also from extrasynaptic sites. Microdialysis studies showed physiological changes in the extracellular levels of serotonin in response to pharmacological or behavioral modulation (Brazell et al., 1985; Sharp et al., 1989; Wright et al., 1992; Pudovkina et al., 2003; Mansari et al., 2007), and voltammetry studies have measured increases in extracellular serotonin upon electrical stimulation (Bunin and Wightman, 1998; Swanson et al., 2005).

The presence of extrasynaptic 5-HT receptors in the central nervous system further supports the possibility of serotonin acting through paracrine or volume transmission (Bunin and Wightman, 1999). 5-HT1A receptors are located exclusively in the somata and dendrites of serotonergic neurons in the dorsal raphe (Kia et al., 1996; Riad et al., 2000), and in non-serotonergic neurons in the hippocampus, suggesting that they modulate neuronal firing of serotonergic and non-serotonergic neurons. On the other hand, 5-HT1B receptors are preferentially associated with preterminal axons in the globus pallidus and the substantia nigra, where they could modulate axonal impulse conduction (Riad et al., 2000). In addition, direct evidence of the extrasynaptic localization of functional 5-HT transporters along axons has been provided by immunohystochemistry in the cingulated cortex, cingulum bundle, medial forebrain bundle, corpus callosum, and dorsal raphe (Zhou et al., 1998). The extrasynaptic serotonin concentrations, as measured by voltammetry, match the affinities of the predominant receptors in each brain region (Bunin and Wightman, 1998), further supporting that serotonin acts through volume transmission.

### Direct evidence and mechanisms of somatic serotonin exocytosis

**Figure 1:**
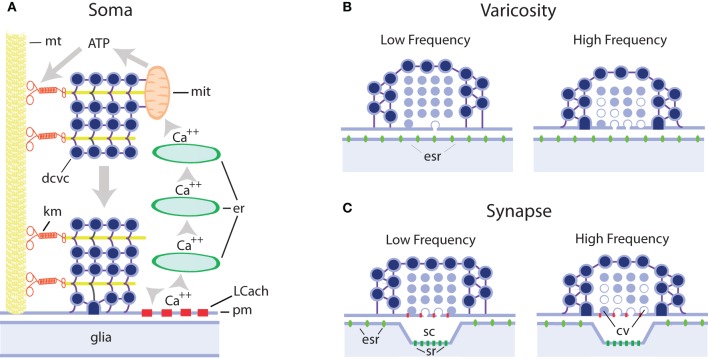
**Schematic representation of extrasynaptic and synaptic exocytosis from clear and dense core vesicles in different regions of serotonergic neurons. (A)** Mechanism of long-lasting somatic secretion from dense core vesicles in leech Retzius neurons. Electrical stimulation of neurons with trains of 10–20 Hz produces transmembrane calcium entry through L type channels (LCach). This calcium in turn activates calcium-induced calcium release from progressively more internal endoplasmic reticulum stores (er). Calcium waves propagate to internal regions of the soma and activate ATP synthesis by the mithocondria (mit). The ATP increase activates kinesin motors (km) that transport dense core vesicle clusters (dcvc) along microtubule rails (mt) towards the plasma membrane (pm), with which they fuse. This exocytosis lasts for minutes after the end of the train of electrical activity. Serotonin is released onto glial cells. **(B)** Hypothetical model of serotonin release from axonal varicosities containing clear and/or dense core vesicles. Both vesicle types may contain serotonin, as in serotonergic raphe neurons, and at rest lay at nanometer distances from the plasma membrane. In these cases exocytosis occurs in the absence of a postsynaptic counterpart and serotonin activates extrasynaptic receptors (esr). It is expected that with this configuration single impulses or low frequency trains do not evoke significant amounts of exocytosis although they contribute to approaching vesicles toward the plasma membrane, thus increasing the probability of release upon subsequent impulses. At high frequencies, both vesicle pools increase their fusion probability. The vesicles with white centers represent those during or after exocytosis **(C)** Synaptic and extrasynaptic exocytosis from synaptic bouttons. Clear vesicles at presynaptic endings are apposed to the plasma membrane and single impulses evoke exocytosis with mechanisms similar to those in neuromuscular junction. Dense core vesicles surrounding clear vesicles have low release probability and subsequent impulses increase the probability of exocytosis. At high frequencies, the clear vesicle pool enters the facilitation/depression dynamics, whereas the dense core vesicle pool increases its release probability. While clear vesicle contents are released onto the synaptic cleft and affect mostly synaptic receptors (sr), dense core vesicles release their contents extrasynaptically and serotonin activates extrasynaptic receptors (esr). For additional details and references see the text.

Direct evidence of physiological somatic exocytosis of serotonin first came from Retzius neurons of the leech central nervous system (Trueta et al., 2003). The soma of these neurons contains “astronomical quantities” of large (100 nm) dense-core vesicles containing serotonin (Coggeshall, 1972). Retzius neurons have the advantage that they can be studied in the ganglion or in culture, since in spite of being adult, they can be isolated individually and maintained in culture for weeks, where they keep their electrical properties, continue to synthesize and release quantal packages of serotonin both from the soma or from synapses with another Retzius neuron or with pressure-sensory neurons (for review see Fernández-de-Miguel and Drapeau, 1995). These synapses were the first established in culture (Fuchs et al., 1981) and have been useful to study on one hand, the steps in the formation of synapses. For example, the formation of serotonergic synapses produces retrograde and anterograde effects on calcium currents and reduces neurite extension by postsynaptic neurons (Cooper et al., 1992; Fernandez-De-Miguel et al., 1992). On the other hand this preparation has allowed studying the fine mechanisms of serotonin exocytosis. In these synapses clear synaptic vesicles are surrounded by electrodense vesicles, and both types of vesicles contain serotonin (Kuffler et al., 1987; Bruns and Jahn, 1995). Serotonin exocytosis from clear and dense core vesicles is quantal (Henderson et al., 1983; Bruns and Jahn, 1995) and calcium dependent (Dietzel et al., 1986), although serotonin release from clear vesicles occurs in response to single action potentials, whereas exocytosis from electrodense vesicles occurs upon subsequent stimulation (Bruns and Jahn, 1995; see Figure 1). Released serotonin reduces the amplitude of action potentials arriving at the serotonergic terminals, thus producing a presynaptic auto-inhibition (Cercós et al., 2009). Altogether, these studies have provided most of our current knowledge about the fine mechanisms of serotonin release from synapses (Nicholls and Kuffler, 1990; Zimmermann, 1993).

Retzius neurons have also provided substantial evidence about the mechanisms of somatic exocytosis of serotonin (De-Miguel and Trueta, 2005; Figure 1). This information is proving useful to explain processes that also apply to other cell types releasing different signaling molecules, as will be seen below.

Addition of ionomycin to the soma of isolated Retzius neurons evokes quantal serotonin exocytosis from dense core vesicles, as demonstrated by amperometric recordings (Bruns et al., 2000). That somatic serotonin exocytosis is physiological was demonstrated by our own studies combining electrophysiology, fluorescence imaging, and electron microscopy in neurons in the ganglion and in culture. Somatic dense core vesicles at rest are located in two pools: one around the nucleus and another in more peripheral areas of the cytoplasm, although distant from the plasma membrane (Rude et al., 1969; Trueta et al., 2004, 2012). Electrical stimulation with trains of 10 impulses at 10 or 20 Hz, but not at a low 1 Hz frequency, induces exocytosis for several minutes. The kinetics of somatic serotonin exocytosis in Retzius neurons have been studied by the gradual incorporation of fluorescent dye FM1-43 (Trueta et al., 2003), which binds to the vesicles as they fuse with the plasma membrane, thus increasing the fluorescence of the dye (Kilic et al., 2001). Exocytosis usually starts tens of seconds after stimulation and lasts for several minutes. The interpretation for this slow kinetics is the involvement of active transport of vesicle clusters from distant intracellular regions to the plasma membrane using microtubules as rails (Trueta et al., 2012; De-Miguel et al., in press). Electrical stimulation activates L-type calcium channels (Trueta et al., 2003), and at high stimulation frequencies, this calcium activates calcium-induced calcium release from intracellular stores (Trueta et al., 2004). It is possible to suppose that calcium arriving at the mitochondria activates ATP synthesis and this in turn activates kinesin-mediated transport of vesicles (Figure 1). An open question is how exocytosis is sustained for such long periods of time. After exocytosis, dense core, and small clear vesicles, the later formed in response to endocytosis, are incorporated into multivesicular bodies that travel back to the perinuclear zone, where they apparently release their contents, some of which are re-used in the formation of new vesicles (Trueta et al., 2012, in this issue).

Each Retzius neuron produces 100–120 FM1-43 fluorescent spots upon a train of ten impulses at 20 Hz (Trueta et al., 2003), each spot corresponding to a release site in which 100–1000 vesicles fuse (De-Miguel et al., in press). Therefore, tens of thousands of somatic vesicles may fuse in response to a 20 Hz train lasting 0.5 s, with each vesicle containing 67,000 molecules of serotonin (Bruns and Jahn, 1995). An equivalent amount of serotonin is secreted by the other Retzius neuron in the ganglion, since both respond in parallel upon the activation of common synaptic inputs (Velázquez-Ulloa et al., 2003). Although several questions still remain open, it is not surprising that a neuron pair is capable of producing the multiple modulatory effects of serotonin seen in the leech (Willard, 1981; Kristan and JrNusbaum, 1982; Burrell et al., 2002; Crisp and Mesce, 2006; Calviño and Szczupak, 2008; Bisson and Torre, 2011).

It is noteworthy that somatic serotonin is released onto glial cells wrapping the somata of Retzius neurons (Trueta et al., 2012). Therefore, not only exocytosis occurs from regions with an apparent absence of well-defined presynaptic structures, but the immediate target is non neuronal. How does serotonin reach its neuronal targets through glial cells is not yet known; however studies on peripheral ATP-releasing somata (Zhang et al., 2007; see below) have provided interesting evidence about this (see below).

A similar slow kinetics of somatic exocytosis of serotonin has been demonstrated in dissociated rat dorsal raphe neurons, by measuring serotonin fluorescence with multi-photon microscopy. Serotonin in the soma of these neurons is contained in perinuclear vesicle clusters and is released upon depolarization with high potassium (Kaushalya et al., 2008a) or glutamate receptor activation (Colgan et al., 2009). The amount of serotonin released from the soma is comparable to that released in areas with a high density of serotonergic processes, suggesting that somatic exocytosis plays a major role in the overall serotonergic transmission (Kaushalya et al., 2008b). Somatic serotonin exocytosis upon stimulation is maintained by continuous vesicular packaging by the vesicular monoamine transporter located in the nucleus and cytoplasm. Serotonin packaged during stimulation is released preferentialy, while that packaged at rest is held in a reserve pool (Colgan et al., 2009).

The frequency dependence of somatic secretion correlates with the responses of 5-HT autoreceptors in raphe nucleus neurons, used as an indirect measure of serotonin release (O'Connor and Kruk, 1991). This frequency dependence and the latency of exocytosis can be explained in terms of a highly regulated secretion process that requires the active transport of vesicles toward the plasma membrane in response to electrical stimulation and, as result of this, high energy expenses (De-Miguel et al., in press). On the other hand a question yet to be answered is how calcium-dependent exocytosis is sustained long after electrical stimulation has ended, since vesicular transport seems to be the major source of delay between stimulation and secretion.

### Regulation of somatodendritic serotonin exocytosis

Somatodendritic serotonin release is regulated by multiple factors, including different synaptic inputs onto serotonergic neurons (Velázquez-Ulloa et al., 2003) and by serotonin itself (see below). In raphe neurons glutamate stimulates somato-dendritic serotonin exocytosis (de Kock et al., 2006; Colgan et al., 2009), and dendritic calcium influx through NMDA receptors evokes exocytosis in the absence of action potentials (de Kock et al., 2006). In contrast, noradrenaline and GABA inhibit serotonin release by activating α-2 and GABA-A receptors, respectively (Kalsner and Abdali, 2001; de Kock et al., 2006).

Somatodendritic serotonin release activates 5-HT2A and 5-TH2C receptors in GABAergic neurons that innervate the dorsal raphe (Liu et al., 2000), thus increasing the frequency of GABAergic inputs onto 5-HT neurons (de Kock et al., 2006), and producing a negative feedback loop within the nucleus. In addition, serotonin released from the somatodendritic compartments activates 5-HT1A, 5-HT1B, and 5-HT1D autoreceptors, which in turn inhibit serotonin exocytosis (Davidson and Stamford, 1995) through the activation of calcium/calmodulin protein kinase II and protein kinase A (Liu et al., 2005).

### Physiological functions of extrasynaptic serotonin release

Important roles for the extracellular elevations of serotonin, presumably from extrasynaptic exocytosis, have been shown in food-related behaviors in *C. elegans* (Harris et al., 2011; Jafari et al., 2011) and leech (Lent and Dickinson, 1984; Lent, 1985; Groome et al., 1993). Serotonin in leeches regulates sensitization of the whole body-shortening reflex by potentiating excitability and modulating the electrical coupling between S-cells (Moss et al., 2005), and induces social behavior (Burrell and Sahley, 2005; see Bisson et al., 2012, in this issue). Circulating serotonin in lobsters determines their social dominance by affecting from sensory inputs to motor outputs of behavioral circuits (Kravitz, 2000), and manipulation of serotonergic transmission in flies modulates fighting and aggression (Alekseyenko et al., 2010), although it has not been determined if these effects operate through synaptic or extrasynaptic mechanisms. Extrasynaptic serotonin may also modulate mood and social behavior in mammals. In fact, the effects of many antidepressant drugs seem to be mostly extrasynaptic (Kiss, 2008). Thus, understanding the regulation of extrasynaptic release of serotonin should lead to improvements in the treatment of depression and other psychiatric disorders.

## Dopamine

From mandibulate fish to mammals (Reiner, 2009), basal ganglia contain the somata of dopaminergic neurons clustered in two main areas: the substantia nigra and the ventral tegmental area (Dahlström and Fuxe, 1964). Substantia nigra neurons project to the striatum of the basal ganglia, forming the nigrostriatal pathway; neurons in the ventral tegmental area project to the nucleus accumbens, amygdala, and prefrontal cortex, forming the mesolimbic and mesocortical systems. The arcuate nucleus and hypothalamus also contain dopaminergic neurons, which project to the median eminence forming the tuberoinfundibular system; dopaminergic neurons in the dorsal raphe nucleus project to the prefrontal cortex (Yoshida et al., 1989).

Dopamine acts exclusively through G-protein-coupled receptors that usually modulate cyclic AMP production (for review, see Romanelli et al., 2010). The D1 family, which includes D1 and D5 receptors, increases the levels of cyclic AMP, whereas activation of D2-like (including D3 and D4) receptors inhibits adenylate cyclase, and thereby decreases the cAMP levels. D1 receptors are located at extrasynaptic sites in dendritic spines in the cerebral cortex of primates (Smiley et al., 1992), while D2 receptors are located both in synaptic terminals and in preterminal axons in the striatum (Sesack et al., 1994). D2 receptors are also located in the somatodendritic area of dopaminergic neurons in the substantia nigra (Sesack et al., 1994), where they produce autoinhibitory effects (see below). The high affinity of D2 receptors suggests that they participate in dopaminergic volume transmission *in vivo* (Marcellino et al., 2011). Dopamine mediates fear and anxiety responses in the amygdala where dopamine release by synaptic terminals produces disinhibition of fear responses, leading to anxious behavior. Extrasynaptic dopamine on the other hand, contributes to reducing the anxiety levels under no danger conditions, by activating GABAergic neurons that inhibit anxiety responses (for review, see Pérez de la Mora et al., 2008).

### Extrasynaptic dopamine exocytosis

As in the serotonergic system, small numbers of dopaminergic neurons give rise to hundreds of thousands of varicosities that diffusely innervate large target areas (reviewed by Descarries and Mechawar, 2000). Dopaminergic varicosities may contain small clear or large dense-cored vesicles but they lack postsynaptic specializations in the cortex (Séguéla et al., 1988; Papadopoulos et al., 1989; Smiley et al., 1992; Smiley and Goldman-Rakic, 1993; Erickson et al., 2000), striatum (Descarries et al., 1996), and spinal cord (Ridet et al., 1993), suggesting that these structures display extrasynaptic exocytosis.

The somata and dendrites of dopaminergic neurons in the substantia nigra and the ventral tegmental area also release dopamine (Björklund and Lindvall, 1975; Geffen et al., 1976; Cheramy et al., 1981; Jaffe et al., 1998), as first shown by H^3^-incubated nigral slices (Geffen et al., 1976), and by push-pull cannula *in vivo* in anaesthetized rats (Nieoullon et al., 1977). More recently, extracellular dopamine increases were detected upon electrical stimulation by use of fast scan cyclic voltammetry in slices containing the substantia nigra or the ventral tegmental area (Rice et al., 1997; Chen and Rice, 2001; Chen et al., 2006; Ford et al., 2010), suggesting that dopamine had been released from the somata and dendrites, presumably through exocytosis (Fortin et al., 2006; Witkovsky et al., 2009). As with varicose axonal endings, the evidence for pre- or postsynaptic specializations in the midbrain DA neurons is limited (Wilson et al., 1977; Groves and Linder, 1983), suggesting that dopamine exocytosis from these compartments is extrasynaptic.

Direct records of dopamine somatic exocytosis have been obtained using carbon fiber amperometry from giant dopaminergic neurons of the pond snail *Planorbis corneus.* These neurons produce bursts of quantal release from two vesicle populations (Chen et al., 1996; Anderson et al., 1999). Amperometric currents recorded from neurons in the pars compacta of the substantia nigra demonstrated quantal release in response to glutamate or high potassium application (Jaffe et al., 1998). This evidence has been complemented by the amperometric demonstration that dopamine is released in the retina from the soma of amacrine cells, depending on electrical activity and calcium entry though L-type channels (Puopolo et al., 2001).

### Mechanisms of extrasynaptic exocytosis of dopamine

That dopamine extrasynaptic release occurs through exocytosis in nigral dopaminergic neurons was supported by its blockade by botulinum toxins (Fortin et al., 2006). However, its mechanisms differ from those of presynaptic terminals in several respects. First, the presynaptic proteins synaptotagmin 1, syntaxin1, synaptic vesicle proteins 2a and 2b, synaptophysin, and synaptobrevin 1 (VAMP 1) are absent from these neurons (Witkovsky et al., 2009). Similar striking results have been obtained from hypothalamic neurons (reviewed by Tobin et al., 2012, in this issue). Second and similar to serotonergic somatic secretion (Trueta et al., 2004), dopamine release in the substantia nigra has a significant contribution of calcium release from intracellular stores (Patel et al., 2009). Third, the time course of evoked increases in extracellular dopamine in guinea pig slices is ten-fold slower in somatodendritic areas in the substantia nigra, than in the synaptic striatal areas (Chen and Rice, 2001). Additional differences have been found in dopaminergic neurons of guinea pig substantia nigra, which has a much lower calcium dependence, with a maximum at 1.5 mM extracellular concentration (Chen and Rice, 2001; Chen et al., 2011), showing high calcium affinity but low cooperativity, while release in the striatum requires concentrations above 1.5 mM and has low affinity and high cooperativity (Chen et al., 2011). While synaptic dopamine release from the striatum is reduced by blockers of N, P/Q, T, or R calcium channels, release in the substantia nigra is insensitive to all these blockers, although it depends on the activation of voltage-gated calcium channels in the substantia nigra of rat (Jaffe et al., 1998) and guinea pig (Patel et al., 2009). These last differences could be a particularity of guinea pig dopaminergic neurons since in mouse brain slices the calcium-dependence and time course of release are similar in the substantia nigra, the ventral tegment, and striatum (Ford et al., 2010).

### Regulation of somatodendritic dopamine release in the substantia nigra

Activation of the serotonergic innervation to the substantia nigra increases somatodendritic dopamine release. In contrast, synaptic dopamine release from the projections of these same neurons to the striatum is not affected by serotonin, suggesting that the serotonergic regulation of somatic release is independent of impulse generation (Cobb and Abercrombie, 2003). Both axonal and somatodendritic release of dopamine are auto-inhibited by dopamine through D2 receptors (Cobb and Abercrombie, 2003).

### Extrasynaptic exocytosis of dopamine in the retina

Dopamine in the retina is produced by a group of amacrine cells that send fine processes toward photoreceptors, bipolar, and horizontal cells (Dacey, 1990; Kolb et al., 1990; Marshak, 2001). However, most of these processes do not make morphologically defined synapses (Bjelke et al., 1996), and although certain amacrine cells receive dopaminergic synaptic inputs (Kolb et al., 1990), the vast majority of retinal cells respond to dopamine that reaches them through volume transmission (Bjelke et al., 1996; Witkovsky, 2004). Dopaminergic amacrine cells release dopamine by exocytosis from the cell body (Puopolo et al., 2001) depending on the cell's electrical activity. This exocytosis is mediated by calcium entry though L-type channels, modulated by glutamate and GABA and auto-inhibited by dopamine through D2 receptors (Puopolo et al., 2001), with this last effect being similar to its autoinhibitory effects in the substantia nigra and striatum (Cobb and Abercrombie, 2003).

In the retina, 50% of the released dopamine escapes transporter uptake by dopaminergic neurons and therefore the dopamine concentration in the extracellular fluid reaches 100–1000 nM concentrations (Witkovsky et al., 1993), high enough to activate dopamine receptors in the different retinal neuronal types (Cohen et al., 1992; Muresan and Besharse, 1993; Nguyen-Legros et al., 1997), in Muller glial cells (Biedermann et al., 1995) and in the pigment epithelium (Versaux-Botteri et al., 1997). Cells that are more distant from dopaminergic amacrine cells have the most sensitive receptors, thus being compatible with volume transmission actions (Witkovsky, 2004).

Retinal dopamine effects that may be attributed to extrasynaptic exocytosis have long been known in the retina. Electrical coupling of horizontal cells allow the flow of light responses of a particular spot to the surrounding cells (Naka and Rushton, 1967; Kaneko, 1971), thus enlarging the horizontal cell's receptive fields at the expense of space resolution. Dopamine reduces the horizontal cell coupling, thus narrowing their receptive fields (Piccolino et al., 1984; Teranishi et al., 1984) and increases the coupling between cone and rod photoreceptors, thus allowing the rod pathway to convey red chromatic information (Krizaj et al., 1998). Dopamine also activates calcium-dependent chloride channels in rods, thus reducing their synaptic transmission onto horizontal and bipolar cells and decreasing the rod pathway output. On the other hand, dopamine increases the cone output by exerting opposite effects on red cones (Stella and Thoreson, 2000; Thoreson et al., 2002). By modulating synaptic transmission from photoreceptors to horizontal and bipolar cells, dopamine changes the balance between rod and cone inputs to second order cells in the retina. Since the production of dopamine in the retina varies with a circadian rhythm, it produces an oscillation between rod and cone dominance depending on the time of the day (Wang and Mangel, 1996; Manglapus et al., 1999).

## Noradrenaline

Noradrenaline in the central nervous system modulates alertness, arousal, reward (Aston-Jones and Cohen, 2005), stress (Valentino and Van Bockstaele, 2008), and sensory information processing (Svensson, 1987). Most noradrenergic neurons have their somata in the locus coeruleus, a pontine nucleus near the fourth ventricle in the brainstem, and they innervate large areas of the nervous system, including the cortex, hippocampus, amygdala, hypothalamus, striatum, thalamus, and spinal cord (Swanson and Hartman, 1975). Like in serotonergic and dopaminergic neurons, axonal projections of noradrenergic neurons have varicose nerve endings, most of which lack synaptic contacts, but seem suitable for extrasynaptic exocytosis. Nearly all noradrenergic varicosities in the cerebral cortex are non-synaptic (Descarries et al., 1977; Aoki et al., 1998), as are more than 60% of those in the hippocampus (Hökfelt, 1968; Umbriaco et al., 1995) and in the dorsal horns of the spinal cord (Ridet et al., 1993).

Noradrenaline acts on metabotropic receptors, many of which are extrasynaptic. In monkey prefrontal cortex, immunocytochemical electron microscopy has revealed that α2A receptors are mostly localized at preterminal axons, dendritic shafts, and astrocytic processes, all lacking morphologically identifiable synaptic junctions (Aoki et al., 1998). α 2A receptors are also located in extrasynaptic sites of dendrites in the central nucleus of the amygdala (Glass et al., 2002) and the tractus solitarius (Glass et al., 2001). β2 adrenergic receptors are also located in extrasynaptic dendritic and preterminal axonal sites in the spinal cord (Mizukami, 2004). Moreover, the presence of dopamine-β-hydroxylase (the noradrenaline synthesizing enzyme) in the soma and dendrites in the locus coeruleus, and the measurement of extracellular noradrenaline by microdialysis correlating with neuronal activity within the nucleus (Reviewed by Singewald and Philippu, 1998; Berridge and Abercrombie, 1999), have suggested that noradrenaline can be released from somatodendritic compartments of locus coeruleus neurons.

Carbon fiber amperometry and capacitance measurements of noradrenaline somatic exocytosis from locus coeruleus neurons (Huang et al., 2007) have demonstrated that, as for serotonergic and dopaminergic neurons, somatic exocytosis occurs from dense core vesicles, can be elicited by depolarization or impulse trains, requires calcium entry and depends on the stimulation frequency. In addition, the >100 ms latency of evoked exocytosis suggests that it also requires vesicle mobilization from internal regions to the plasma membrane.

Noradrenaline somatic exocytosis in the locus coeruleus is stimulated by NMDA and by the hormone hypocretin, which potentiates the NMDA-mediated exocytosis through the activation of protein kinase C (Chen et al., 2008). Somatic exocytosis of noradrenaline autoinhibits noradrenergic neurons through the activation of α 2a adrenoreceptors, which produces a potassium-dependent hyperpolarization and decreases their firing rate (Williams et al., 1985). This in turn decreases noradrenaline release both in the locus coeruleus (Pudovkina et al., 2001; Fernández-Pastor et al., 2005; Huang et al., 2007) and in the brain projections of these neurons (Fernández-Pastor and Meana, 2002). The frequency-dependence of somatic noradrenaline exocytosis suggests that stress-induced hyperactivity of locus coeruleus neurons autoinhibits these neurons and prevents possible damaging effects of extracellular noradrenaline excess in epilepsy, stress disorders, or disorders in the sleep/arousal cycles (Reviewed by Huang et al., 2012).

## Acetylcholine

Acetylcholine in the brain mediates sophisticated central aspects of behavioral control, including wakefulness and somnolence the readiness of the forebrain for input processing and essential aspects of attentional information processing (Yu and Dayan, 2002; Sarter et al., 2006; Parikh et al., 2007). The whole cerebral cortex and the hippocampus are innervated by cholinergic neurons originating in the nucleus basalis of Meynert, the substantia innominata, and the horizontal limb of the diagonal band. Cholinergic neurons in the tegmental area innervate the thalamus and midbrain dopaminergic areas, while neurons originating in the medial habenular nucleus form the habenulo-interpeduncular tract, and neurons in the striatum innervate this area and the ofactory tubercle.

In contrast to monoaminergic neurons that contain clear and dense core vesicles, cholinergic neurons in the cerebral cortex, hippocampus, and striatum have axonal varicosities bearing only small clear vesicles (Descarries and Mechawar, 2000). However, like in serotonergic and dopaminergic systems, some of these structures are synaptic (Smiley et al., 1997; Turrini et al., 2001) but most are not (Umbriaco et al., 1994; Kasa et al., 1995; Descarries et al., 1997; Descarries and Mechawar, 2000), suggesting that extrasynaptic exocytosis also takes place in the cholinergic system. In support of this morphological evidence, acetylcholine has been measured in the extracellular space by microdialysis in the striatum and the cortex (Fadel et al., 1996; Vinson and Justice, 1997; Johnson et al., 2012) showing level variations during different arousal states (reviewed by Sarter et al., 2009).

Most cholinergic receptors in the central nervous system are metabotropic and thus mediate slow indirect responses characteristic of volume transmission, although central extrasynaptic locations also contain ionotropic nicotinic receptors (reviewed by Vizi et al., 2010). Metabotropic muscarinic M1 and M2 receptors, with high affinity, are located in cortical non-cholinergic synapses (Mrzljak et al., 1993), suggesting that acetylcholine diffuses to reach these heterosynaptic receptors.

Immunolabeling or α-bungarotoxin binding in electron microscopy sections demonstrate nicotinic receptors in extrasynaptic sites or in pre- or post-synaptic terminals of GABAergic or glutamatergic synapses in the ventral tegmental area (Jones and Wonnacott, 2004), the hippocampus (Vizi and Kiss, 1998; Fabian-Fine et al., 2001), and the chick ciliary ganglion (Jacob and Berg, 1983), where they modulate the release of transmitters and their postsynaptic effects (Lendvai and Vizi, 2008). The dendrites of bipolar, amacrine, and ganglion cells in the goldfish retina also have nicotinic acetylcholine receptors in non-synaptic sites (Zucker and Yazulla, 1982). In addition, nicotinic receptors are expressed by microglial cells (Shytle et al., 2004) and astrocytes (Gahring et al., 2004), further supporting the possibility that acetylcholine diffuses through the extracellular fluid and regulates neuronal and glial cell activity. The presence of extrasynaptic nicotinic receptors suggests that they produce faster volume transmission responses than those mediated by metabotropic receptors for biogenic amines (for a detailed review, see Lendvai and Vizi, 2008).

Acetylcholine was one of the first transmitters found to be released from neuronal somata. Calcium-dependent acetylcholine somatic release in response to depolarization with high potassium or to antidromic electrical stimulation of ciliary nerves was found in denervated parasympathetic ganglia incubated with [^3^H]-choline (Johnson and Pilar, 1980). In the habenullo-interpeduncular tract, optical stimulation of cholinergic neurons expressing ChanelRhodopsin-2 induces co-release of glutamate and acetylcholine from the same terminals. Brief stimulation pulses induce glutamate release which acts as a direct transmitter; tetanic stimulation induces acetylcholine release, which produces slow inward currents in the interpeduncular nucleus cells, mediated by nicotinic receptors. The slow time course of these currents suggests that acetylcholine acts through volume transmission (Ren et al., 2011).

In autonomic ganglia synapses, the level of acetylcholinesterase activity is lower than that in the neuromuscular junction (Hartzell et al., 1977) and this could allow acetylcholine synaptic spillover. However, at synapses with a high concentration of cholinesterase, such as in the neuromuscular junction, the degradation of acetylcholine after dissociation from its receptors is so efficient, that a single molecule cannot activate a second receptor (Kuffler and Yoshikami, 1975). Thus, the evidence that acetylcholine acts by volume transmission in the brain could be an indication that this transmitter is released also from extrasynaptic sites, where it does not encounter acetylcholinesterase immediately.

Acetylcholine exocytosis from the axon, dendrites, and soma was demonstrated by use of outside-out patches of embryonic muscle membrane as detectors of release from *Xenopus* dissociated spinal neurons (Sun and Poo, 1987). Somatic exocytosis in this preparation requires prolonged suprathreshold stimulation and is dependent on the extracellular calcium concentration, supporting that vesicles need to move toward the plasma membrane. Activity- and calcium-dependent exocytosis from neurite varicosities has been demonstrated by a similar technique in magnocellular neurons dissociated from the rat basal forebrain, where exocytosis is autoinhibited by acetylcholine acting though muscarinic receptors (Allen and Brown, 1996).

## Glutamate

Extrasynaptic glutamate accumulates from extrasynaptic vesicular exocytosis, synaptic spillover and non-vesicular release through reversal of transporters. In neuropathic rats, the down regulation of glial glutamate transporters leads to spillover from peripheral sensory to spinal horn synapses, producing an increased NMDA receptor activation that in turn results in pathological pain (Nie and Weng, 2010). Evidence suggests that glutamate spillover and activation of extrasynaptic receptors occurs when large glutamate amounts are released from synaptic endings. For example, in the cerebellar molecular layer interneurons, activation of small numbers of parallel fibers by short stimuli produces only AMPA receptor-dependent excitatory postsynaptic currents, but synaptic facilitation or activation of large numbers of fibers adds a slow NMDA receptor-mediated current that may have an extrasynaptic origin (Clark and Cull-Candy, 2002; Ikonomu et al., 2012 in this issue).

Extrasynaptic glutamate is co-released with ATP through calcium-dependent vesicular exocytosis from olfactory bulb axons (Rieger et al., 2007; Thyssen et al., 2010) in response to action potentials. Both transmitters mediate axonal-glial communication through activation of mGluR1 and P2Y1 receptors respectively (Rieger et al., 2007). These transmitters also activate calcium signals in white matter astrocytes, which in turn release ATP, thus spreading the calcium signals to neighboring glial cells (Hamilton et al., 2008).

Astrocytes in culture and in slice preparations also release glutamate by several mechanisms (Parpura et al., 1994; Bezzi et al., 1998; reviewed by Malarkey and Parpura, 2008) including calcium-dependent vesicular exocytosis, as demonstrated by use of total internal reflection fluorescence (TIRF) microscopy (Bezzi et al., 2004), amperometry of dopamine as a surrogate transmitter for glutamate (Chen et al., 2005), or measurements of membrane capacitance (Zhang et al., 2004). Exocytosis in astrocytes occurs from small clear vesicles, which fuse with the membrane using the SNARE protein complex. Calcium release from IP3- and ryanodine-sensitive stores has a crucial contribution to this astrocytic glutamate exocytosis (Hua et al., 2004). Although glutamate release sites in astrocytes are unknown, this source of extrasynaptic glutamate can produce calcium elevations in neighboring neurons (Bezzi et al., 1998).

## GABA

GABA is the main inhibitory synaptic transmitter in the nervous system. However, the presence of extrasynaptic GABA receptors in addition to the occurrence of slow and diffuse effects of GABAergic neurons, and the tonic inhibition of neurons in different areas of the central nervous system, suggest that GABA acts also through volume transmission.

Extrasynaptic GABA-A receptors are present in several areas of the nervous system (Somogyi et al., 1989; for review see Mody, 2001; Kullmann et al., 2005). In cerebellar granule cells, the number of extrasynaptic GABA-A receptors is higher than that of synaptic receptors (Nusser et al., 1995). Moreover, GABA-A receptors containing the delta subunit are exclusive of extrasynaptic sites in these cells (Nusser et al., 1998) and in hippocampal dentate gyrus granule cells (Wei et al., 2003). It is noteworthy that these receptors have a 50-fold higher affinity for GABA than other GABA-A receptors, and do not desensitize upon the prolonged presence of agonists (Saxena and Macdonald, 1994), thus being suitable to mediate tonic extrasynaptic inhibition. Extrasynaptic GABA-Aδ receptors in the suprachiasmatic nucleus modulate circadian phase shifts (McElroy et al., 2009). GABAρ receptors in the neostriatum and the hippocampus are perisynaptic and extrasynaptic (Rosas-Arellano et al., 2011a,b).

In agreement with the presence of extrasynaptic receptors, extracellular GABA, as measured by microdialysis, is present in the extracellular space of rat hippocampus (Lerma et al., 1986; de Groote and Linthorst, 2007), striatum (Kennedy et al., 2002), and nucleus accumbens (Xi et al., 2003), and extrasynaptic or paracrine effects of GABA occur in the cortex (Zhu et al., 2008; Oláh et al., 2009; Vélez-Fort et al., 2010), cerebellum, and hipocampppus, where stress increases the extracellular GABA levels (de Groote and Linthorst, 2007). The excitability of cerebellar granule cells is reduced by GABA in the extracellular fluid, through the activation of a tonic inhibitory current (Kaneda et al., 1995; Nusser et al., 1998; for review see Farrant and Nusser, 2005). A similar tonic GABAergic inhibition exists in dentate gyrus granule cells, thalamocortical relay neurons of the ventral basal complex (Porcello et al., 2003), CA1 pyramidal neurons (Bai et al., 2001), inhibitory interneurons in the CA1 region of the hippocampus (Semyanov et al., 2003), neurons of the medial geniculate body of the auditory thalamus (Richardson et al., 2011), neurons in the avian nucleus laminaris (Tang et al., 2011), and in the neuromuscular junction of crustaceans (Parnas et al., 1975). In the avian nucleus laminaris, this GABA-A receptor-mediated tonic inhibition improves coincidence detection by sharpening excitatory postsynaptic potentials and reducing spike probability, thus improving the localization of sound by birds (Tang et al., 2011).

In the cerebellum, the extrasynaptic GABA producing this tonic inhibition during development is released by exocytosis from Golgi cells (Tia et al., 1996). During cerebellar development, GABA is located in all regions of Golgi cells and the vesicular GABA transporter accumulates in axon varicosities and growth cones, suggesting that GABA is released from these structures (Takayama and Inoue, 2004). In the adult, however, extrasynaptic GABA apparently comes both from vesicular (Brickley et al., 2003) and non-vesicular release (Rossi et al., 2003) and it is not clear if release occurs from extrasynaptic sites or if GABA increases upon synaptic spillover. In the hippocampus, tonic (extrasynaptic) and phasic (synaptic) inhibition change in a correlated manner, suggesting that the source of GABA is the same for both effects and that tonic inhibition could have a synaptic spillover origin (Glykys and Mody, 2007).

Extrasynaptic GABA release has been demonstrated in cortical neurogliaform interneurons (Oláh et al., 2009), which form dense axonal arborizations with varicosities that establish very few synapses, as shown by 3D reconstructions obtained from serial electron micrographs. Despite the low incidence of synaptic contacts, stimulation of these interneurons causes slow inhibitory potentials in most of their surrounding cells, suggesting that transmission takes place through GABA diffusion. In addition, stimulation of neurogliaform cells decreases the synaptic responses of other cells in the circuit only if applied at least 120 ms before stimulation of a presynaptic neuron, indicating a volume effect.

Direct evidence of extrasynaptic vesicular co-release of GABA and dopamine comes from the soma of dissociated amacrine retinal cells. Somatic GABA exocytosis is calcium-dependent and produces autocrine miniature-like currents, with variable latencies after stimulation (Hirasawa et al., 2009), suggesting again the fusion of vesicles arriving from different distances to the plasma membrane, as in somatic exocytosis of other signaling molecules (Trueta et al., 2004; Huang et al., 2007; Xia et al., 2009).

## ATP

ATP in neurons is a multifunctional nucleoside, used as energetic “currency” and as a signaling molecule. Extracellular ATP activates ionotropic and metabotropic purinergic P2 receptors, some of which, like P2Y receptors, are extrasynaptic in hippocampal and glial cells (Rodrigues et al., 2005; Hussl and Boehm, 2006). Metabotropic P2 receptors (P2Y, P2U, and P2T) activate phospholipase C through G_q/11_ proteins, or inhibit adenyl cyclase through G_i_ proteins. On the other hand, synaptic ionotropic receptors (P2X and P2Z) allow the transmembrane flux of cations. It is noteworthy that ATP leaks out from neurons through pannexin hemichannles, a way of non synaptic release (Li et al., 2011).

Dorsal root ganglia neurons release ATP by exocytosis from the soma, as shown by the opening of P2X2-EGFP receptors in membrane patches used as biosensors (Zhang et al., 2007). Somatic release is quantal and after stimulation, electron micrographs show photoconverted fluorescent dye FM1-43 inside clear vesicles. ATP exocytosis depends on calcium entry through L-type calcium channels and increasing the stimulation frequency increases the amount of exocytosis and reduces its latency to a minimum of several seconds. Interestingly, somatic ATP exocytosis evokes an increase in the intracellular calcium concentration in the satellite glial cells enwrapping the neurons, which respond by releasing the tumor necrosis factor TNFα. This peptide in turn potentiates the neuronal responses to ATP and their excitability, thus triggering a bi-directional neuron-glia communication (Zhang et al., 2007).

Extrasynaptic sites in axons of olfactory bulb and optic nerve neurons co-release ATP and glutamate (Rieger et al., 2007; Hamilton et al., 2008). Astrocytes in the optic nerve become activated by either transmitter, and respond to them by releasing more ATP, which in turn triggers a calcium wave that spreads to neighboring glial cells (Hamilton et al., 2008). The co-release of ATP and glutamate in olfactory bulb axons is calcium-dependent and occurs through vesicular exocytosis, as shown by its elimination in the presence of bafilomycin A1 and botulinum A toxins (Rieger et al., 2007) Calcium signals produced in the ensheathing glial cells by ATP and glutamate mediate neurovascular coupling, resulting in constriction of adjacent blood vessels (Thyssen et al., 2010).

## Peptides

There is now a wide catalog of peptides released by neurons that modulate neuronal activity or act as hormones when secreted to the circulatory system. Peptide transmitters are released by exocytosis from dense core vesicles in the soma, axons, dendrites, and perisynaptic sites in a variety of neurons upon high frequency electrical activity.

Peptide exocytosis from presynaptic active zones or from postsynaptic dendrites modulates the synaptic function in a localized manner. By contrast, when released from extrasynaptic locations, for example the soma or axons, peptides diffuse away, and act through volume transmission (Agnati et al., 1986a,b). Somatic exocytosis from dense core vesicles has been demonstrated by electron microscopy, through the formation of omega figures in sympathetic or hypothalamic neurons (Zaidi and Matthews, 1997, 1999; see Tobin et al., 2012 in this issue). Somatic exocytosis of substance P was also demonstrated in dissociated dorsal root ganglia neurons by capacitance increases in response to high potassium depolarization, and confirmed by single-cell immunoblot assays (Huang and Neher, 1996). In these neurons somatic exocytosis has a calcium-dependence 10 times lower than that of presynaptic exocytosis, but similar to that for dense core vesicle fusion in neuroendocrine cells, which requires more homogeneous calcium levels to promote vesicle transport, but lower calcium concentration for their fusion (Augustine and Neher, 1992; Becherer et al., 2003).

Ultrastructural studies in the trigeminal subnucleus caudalis of rats showed exocytosis of large dense core vesicles in extrasynaptic locations at axon terminals and dendrites containing substance P (Zhu et al., 1986). The immunofluorescence levels of this peptide correlate with the number of extrasynaptic dense core vesicles undergoing exocytosis counted in electron micrographs after lessoning the whisker area, suggesting that substance P exocytosis mediates neuronal responses to lesions.

The soma of neurons in the supraoptic nucleus releases oxytocin by exocytosis, as shown by omega figures in electron microscopy studies (Morris and Pow, 1991) and by capacitance increases after stimulation (Soldo et al., 2004). Exocytosis depends on the magnitude and duration of the depolarization or on action potential firing at frequencies higher than 13 Hz. Physiological electrical activity patterns, such as those recorded during the milk ejection reflex, stimulate this exocytosis. Notably, oxytocin activates its own somatodendritic exocytosis through activation of G protein-coupled receptors that in turn trigger calcium release from intracellular stores (Lambert et al., 1994), thus producing self-sustained and long lasting exocytosis (Ludwig and Leng, 2006). Somatic exocytosis of oxytocin from these neurons is also induced by NMDA receptor activation (de Kock et al., 2004) and this stimulation is up-regulated during lactation. It is also noteworthy that extrasynaptic ATP in this nucleus facilitates glutamate release (Vavra et al., 2011) and by doing so potentiates oxytocin somatic exocytosis. In turn, oxytocin released in this way inhibits GABA release from presynaptic terminals contacting oxytocinergic neurons, thus removing inhibition and providing a positive feedback for release.

Somatic exocytosis from dense core vesicles has also been directly shown in cultured hippocampal neurons using fluorescent propeptide cargo and TIRF microscopy. Exocytosis events were triggered by calcium entry with long time constants of 16 s and showed rapid fusion-pore openings and closures (kiss and run), associated with limited cargo secretion (Xia et al., 2009). These long latencies in exocytosis from dense core vesicles are also characteristic of somatic exocytosis of other transmitters, as shown in previous sections.

Hypothalamic neurons secreting gonadotropin releasing hormone (GnRH) in a pulsatile way, take up the fluorescent dye FM1-43 by endocytosis in the soma, dendrites, and axon in response to electrical activity. GnRH and vesicle-associated membrane protein (VAMP) co-localize in double immunocytochemistry images, confirming somatic vesicular hormone release, which might contribute to synchronizing electrical activity among GnRH neurons (Fuenzalida et al., 2011).

Neurotrophins such as nerve growth factor (NGF) and BDNF are also released by exocytosis from dense core vesicles in different areas of the nervous system and regulate neuronal survival (Levi-Montalcini and Hamburger, 1953) and synaptic plasticity (for review see Lu et al., 2005). Although neurotrophin secretion *in situ* has been studied by measuring the bulk secretion in the extracellular medium, NGF release occurs from the soma and dendrites of cultured hippocampal neurons (Blöchl and Thoenen, 1996). Neurotrophin release requires stimulation in bursts of high frequency impulses (Balkowiec and Katz, 2000; Lever et al., 2001) and calcium release from intracellular stores following transmembrane calcium entry through voltage-gated channels (reviewed by Lessmann et al., 2003).

Neurons in the mesencephalic trigeminal nucleus display somatic exocytosis from small clear vesicles (Zhang et al., 2012) with a calcium and electrical activity dependence. However, it is not clear what molecules these neurons release.

## Concluding remarks

Increasing evidence is firmly establishing that different signaling molecules are released by exocytosis from extrasynaptic sites in different types of central and peripheral neurons of vertebrates and invertebrates. The mechanisms of extrasynaptic exocytosis are significantly different from those of exocytosis from synaptic terminals (see Figure 1), and share similarities with the mechanisms of exocytosis from excitable endocrine cells. While central synaptic terminals usually release small amounts of quanta of neurotransmitters, enough to produce local hard-wired responses, the large amounts of molecules necessary for volume transmission may be reached by exocytosis from extrasynaptic locations in the cell body, axons, and dendrites.

Studies of exocytosis in the neuronal soma, which allows direct electrophysiological, amperometric and optical recordings of exocytosis and calcium concentrations, which can be correlated with ultrastructural analysis, have shown that somatic exocytosis occurs from small clear vesicles or large dense core vesicles. The volume of dense core vesicles allows them to pack about 15 times more transmitter than small clear vesicles and therefore to produce larger extracellular transmitter increases upon exocytosis of single quanta. In most extrasynaptic exocytosis sites vesicles rest at distances from the plasma membrane ranging from nanometers in varicosities to several microns in the somata. The need for vesicle transport (or diffusion) as an intermediate step in the excitation-secretion coupling confers long latencies and time courses to exocytosis. In addition, the requirement of high stimulation frequencies is explained by the need of intracellular calcium waves produced by transmembrane calcium flow through L-type calcium channels, which are resistant to inactivation and therefore efficient for calcium injection under subsequent depolarizations, followed by the activation of calcium release from intracellular stores. These intracellular changes in calcium concentration are required for exocytosis, but in leech serotonergic neurons also seem to trigger the active vesicle transport toward the plasma membrane by using cytoskeletal-coupled molecular motors. The known mechanisms involved in extrasynaptic exocytosis in different preparations discussed in this review have been summarized in Table 1.

**Table 1:** **Mechanisms for extrasynaptic exocytosis in different cell types**.

**Signaling molecule**	**Subcellular compartment**	**Preparation**	**Vesicle type**	**Resting distance from vesicles to plasma membrane**	**Trans-membrane calcium channel type triggering exocytosis**	**Intracellular calcium sources**	**Latency to beginning of exocytosis**	**Duration of exocytosis**	**Exocytosis-triggering stimuli**	**Recording techniques and references**
Serotonin	Soma	Leech Retzius neurons	Large dense core	>600 nm	L-type	Ryanodine-sensitive	Seconds to minutes	2–10 min (after 0.5 s train)	Long depolarizations, trains at 10 or 20 Hz	Amperometry (Bruns et al., 2000); FM1-43; Electron microscopy (Trueta et al., 2003, 2012).
	Soma	Rat raphe neurons	NA	NA	NA	NA	NA	Minutes	Long depolarizations	3-photon microscopy (Kaushalya et al., 2008a)
Dopamine	Soma	Giant snail dopaminergic neurons	Large dense core	NA	NA	NA	NA	Seconds	Long depolarizations	Amperometry (Chen et al., 1996)
	Soma	Neurons in substantia nigra slices	NA	NA	NA	Ryanodine- and IP3-sensitive	Seconds	NA	Long depolarizations, application of glutamate or serotonin	Amperometry (Jaffe et al., 1998)
	Soma	Retinal amacrine cells	Small clear and large dense core	NA	L-type	NA	NA	Seconds	Long depolarizations; trains at 20 Hz	Amperometry (Puopolo et al., 2001)
Noradrenaline	Soma	Locus coeruleus slices	Large dense core	~200 nm	NA	NA	>100 ms Avg = 1,870 ms	Seconds	Long depolarizations; trains at >20 Hz frequencies; NMDA or hypocretin application	Amperommetry and capacitance records (Huang et al., 2007)
Acetylcholine	Soma and neurites	Xenopus cultured spinal cord neurons	small clear	NA	NA	NA	1–40 ms (1–5 ms for burst events)	2–17 ms	Long depolarizations; trains at 30 Hz	Sniffer patches as sensors (Sun and Poo, 1987)
Glutamate	Soma	Astrocytes in brain slices	small clear	NA	NA	Ryanodine and IP3-sensitive	NA	Seconds	Calcium oscillations; ionomicin application; caged calcium photolysis	Total internal refraction fluorescence microscopy (Bezzi et al., 2004), capacitance measurements (Zhang et al., 2004); amperometry (Chen et al., 2005)
	Axon	Olfactory bulb	NA	NA	NA	NA	NA	NA	Trains at 20 Hz	Calcium signals in ensheating glial cells in response to stimulation of olfactory nerve axons (Rieger et al., 2007)
GABA	Soma and dendrites	Cortical slices	small clear	NA	NA	NA	NA	NA	Single action potentials	Electrophysiological records from cortical neurons upon stimulation of neurogliaform cells (Oláh et al., 2009).
	Soma	Retinal amacrine cells	NA	NA	L-type, R-type	NA	7-960ms	Seconds	1-s depolarizations	Miniature-like currents in retinal amacrine cells (Hirasawa et al., 2009)
ATP	Soma	Dorsal root ganglia neurons	small clear	500 nm	L-type	NA	Seconds	2–5 min	Trains at >20Hz	Sniffer patches expressing P2X2 as sensors and capacitance measurements (Zhang et al., 2007).
	Axon	Olfactory bulb		NA	NA	Cyclopiazonic acid-sensitive intracellular stores	NA	NA	Trains at 20Hz	Calcium signals in ensheating glial cells; exocytosis blocking toxins (Rieger et al., 2007)
Oxytocin	Soma and dendrites	Supraoptic nucleus neurons	Large dense core	NA	High voltage-activated	Thapsigargin-sensisitve intracellular stores	NA	Seconds	Trains at >13 Hz; Glutamate or Oxytocin application	Omega figures in electronmicrographs (Morris and Pow, 1991). Capacitance measurements (Soldo et al., 2004)

Only direct evidence of extrasynaptic exocytosis is summarized. References are provided in parenthesis. The cases where information is not available are indicated by NA.

The different frequency-dependence of synaptic and extrasynaptic exocytosis allows neurons to select from which compartments transmitter will be released. This possibility makes of these neurons multifunctional units in terms of their transmission possibilities, by contributing to fast transmission in hard wired circuits, and slow transmission through volume communication. This capability is of particular relevance for monoaminergic and cholinergic systems, which are composed of small numbers of neurons clustered in specific nuclei that send projections to large brain areas, and modulate a wide variety of functions. In addition, the auto-inhibitory mechanisms of transmitters, along with the multiple steps that couple excitation with exocytosis make of extrasynaptic exocytosis a highly regulated process.

Another feature of extrasynaptic exocytosis is that transmitters may be released directly onto glial cells, which seem to act as mediators for communication with other neurons and with other cells such as those in blood vessels. Therefore extrasynaptic exocytosis of transmitter molecules allows a wide variety of modulatory and integrative properties in the nervous system, from the local modulation of synaptic function to a wide modulation of whole brain areas.

### Conflict of interest statement

This research was conducted in the absence of any commercial or financial relationships that could be construed as a potential conflict of interest.
